# The effect of mother and newborn early skin-to-skin contact on initiation of breastfeeding, newborn temperature and duration of third stage of labor

**DOI:** 10.1186/s13006-018-0174-9

**Published:** 2018-07-16

**Authors:** Kolsoom Safari, Awaz Aziz Saeed, Shukir Saleem Hasan, Lida Moghaddam-Banaem

**Affiliations:** 1 Department of Nursing, College of Nursing, Hawler Medical University, Erbil, Iraq; 2 Department of Midwifery and Reproductive Health, Faculty of Medical Sciences, Tarbiat Modares University, Tehran, Iran

**Keywords:** Early skin-to-skin contact, Temperature, Third stage of labor, Initiation of breastfeeding

## Abstract

**Background:**

Mother and newborn skin-to-skin contact (SSC) after birth brings about numerous protective effects; however, it is an intervention that is underutilized in Iraq where a globally considerable rate of maternal and child death has been reported. The present study was conducted in order to assess the effects of SCC on initiation of breastfeeding, newborn temperature, and duration of the third stage of labor.

**Methods:**

A quasi-experimental study was conducted on 108 healthy women and their neonates (56 in the intervention group who received SSC and 52 in the routine care group) at Hawler maternity teaching hospital of Erbil, Iraq from February to May, 2017. Data were collected via structured interviews and the LATCH scale to document breastfeeding sessions.

**Results:**

The mean age of the mothers in the SSC and routine care groups were 26.29 ± 6.13 (M ± SD) and 26.02 ± 5.94 (M ± SD) respectively. Based on the LATCH scores, 48% of mothers who received SSC and 46% with routine care had successful breastfeeding. Newborns who received SSC initiated breastfeeding within 2.41 ± 1.38 (M ± SD) minutes after birth; however, newborns who received routine care started breastfeeding in 5.48 ± 5.7 (M ± SD) minutes. Duration of the third stage of labor in mothers who practiced SSC after birth was 6 ± 1.7 min, compared to 8.02 ± 3.6 min for mothers who were provided with routine care (*p* <  0.001). Moreover, the prevalence of hypothermia in the newborns who received SSC and routine care was 2 and 42% respectively. Results remained unchanged after using regression modelling to adjust for potential factors and background characteristics.

**Conclusion:**

Skin-to-skin contact provides an appropriate and affordable yet high quality alternative to technology. It is easily implemented, even in small hospitals of very low-income countries, and has the potential to save newborns’ and mothers’ lives. It is necessary to prioritize training of health providers to implement essential newborn care including SSC. Community engagement is also needed to ensure that all women and their families understand the benefits of SSC and early initiation of breastfeeding.

**Trial registration:**

ClinicalTrials.gov: NCT03548389.

## Background

The rate of maternal and neonatal mortality is unacceptably high in Iraq. The maternal and neonatal mortality rates are as high as 84 cases per 100,000 live births and 23 cases per 1000 live births, respectively [1]. These figures are significantly higher in Iraq than developed countries, for example neonatal mortality rates was reported to be 1.74 per 1000 birth in the UK in 2015 [2]. Perinatal infections and fetal hypoxia are the most important causes of neonatal deaths in Iraq, which can be avoided through early initiation of exclusive breastfeeding [1]. However, the prevalence of early initiation of breastfeeding in Iraq is quite low at 38.1% [3].

As recommended by the Baby Friendly Hospital Initiative (BFHI), newborn infants should be placed in skin-to-skin contact with their mothers immediately after their birth for at least one hour, and mothers should be helped to initiate breastfeeding within the first half-hour following the birth of their infants [4, 5]. The term skin-to-skin contact (SSC) is defined as the placement of a naked infant, occasionally with a diaper or a cap on, on its mother’s bare skin, and the exposed side/back of the infant covered by a blanket or a towel [6]. The movement of the infant’s hands over the mother breasts during SSC leads to increased secretion of oxytocin, which results in increased secretion of breast milk [7].

It is also known that SSC after birth promotes newborn temperature regulation, metabolic adaptation, and maintenance of glucose blood levels. Infants have a reduced capacity to generate heat, which leads to a rapid decline in temperature. This is why maintenance of temperature is one of the most important needs of infants at birth [8]. While the mother and her infant are in SSC, heat is transferred from the mother to the infant, during which the mother’s body temperature activates the infant’s sensory nerves, which in turn results in the infant’s relaxation, reduction in the tone of the sympathetic nerves, dilation of skin vessels, and increase in its temperature [7]. Hypothermia during the newborn period is widely regarded as a major contributory cause of significant morbidity and, at its extreme, mortality in developing countries [9]. High prevalence of hypothermia has been reported in countries with the highest rate of neonatal mortality, where hypothermia is increasingly gaining attention and significance as a critical intervention for newborn survival [10].

In addition providing the newborn with numerous benefits, SSC is associated with many benefits for mothers. Secretion of maternal oxytocin in mothers who receive SSC strengthens uterine contractions, which in turn helps the placenta to separate and the duration of the third stage of labor to decrease [11]. The third stage of labor, which involves separation and expulsion of the placenta and membranes, starts immediately after the delivery of the fetus [12]. In most obstetric settings, active management of the third stage of labor is now a common practice to accelerate the third stage, in which synthetic oxytocin that causes the uterus to strongly contract is administered. In a spontaneous, uncomplicated birth, it is reasonable to plan a physiological or natural third stage by utilizing the mother’s own oxytocin [13, 14].

There is an urgent need to reorganize and restructure health services throughout Iraq, and maternity and neonatal care is one of the critical areas that needs substantial efforts in this regard [15]. As a cost-effective, simple and appropriate method, mother and newborn SSC after birth should be practiced in order to improve post-delivery care and potentially save the lives of mothers and newborns [16]. There are very few *studies* that focus on the effects of SSC on maternal and newborn health in Iraq. The present study was carried out in order to determine the effect of early maternal-newborn SSC after birth on the duration of the third stage of labor, early initiation of breastfeeding, and newborn temperature.

To achieve the objectives of the study, the following hypotheses were tested:

1. Mothers who practice early mother and newborn SSC after giving birth experience a shorter duration of the third stage of labor compared with those who do not practice SSC.
2. Mothers who practice early mother and newborn SSC after giving birth exhibit earlier initiation of breastfeeding compared with those who do not practice SSC.
3. Newborns with mother and newborn SSC exhibit normal body temperature 30 min after birth compare with those who do not receive this contact.
4. Mothers who practice early mother and newborn SSC after giving birth exhibit more successful breastfeeding compared with those who do not perform this contact.

## Methods

A quasi-experimental study was conducted on 108 mothers and their neonates in the maternity department of Hawler maternity teaching hospital in Erbil, Iraq from February to May, 2017. Hawler maternity teaching hospital is one of the largest and busiest maternity hospital in Erbil. Erbil is a city that lies 80 km (50 miles) east of Mosul, and is the capital of the Kurdistan Region of Iraq in which the dominant language spoken by residents is Kurdish [17].

Of the 130 women who were eligible to participate in this study, twenty-two women were unable to continue SSC for 1 h after birth, therefore they were excluded. Finally, we included results from 108 mothers in this study who were randomized into two groups: an intervention group consisting of 56 mothers; and the control group consisting of 52 mothers.

Mothers in both groups were homogeneous in terms of their age and gravidity. Laboring women and newborns who met the following conditions were included in the study:

- Normal pregnancy
- Full-term (38 to 42 weeks of gestation)
- Anticipated normal vaginal delivery and desire to breastfeed the infant at birth
- Lack of receiving any pharmacological pain relief
- Willing to join the study
- Newborns with an Apgar score >  7

In this study SSC meant holding the newborn baby undressed in a prone position against the mother’s bare chest between breasts while the back of the baby was covered with a blanket. This SSC commenced immediately after giving birth and continued for 1 h.

### Study instruments

Four instruments were used to collect data. The first instrument was a questionnaire to gather the required demographic and obstetric data from the mothers, including age, gravidity, number of miscarriages, parity, and history of lactation, along with the demographic data of the infants, including weight and gender. The second instrument was a written form that was used to assess the duration of the third stage of labor, which was measured from time of delivery of the infant to the time of complete delivery of the placenta [18]. The third instrument was a written form to record axillary temperatures of the newborns. The fourth instrument was the LATCH breastfeeding assessment tool. LATCH is a sensitive, reliable and valid tool that evaluates breastfeeding techniques based on observations and descriptions of effective breastfeeding [19, 20]. The letters of the acronym LATCH designate five separate assessment parameters: “L” for how well the infant latches onto the breast, “A” for the amount of audible swallowing, “T” for the mother’s nipple types, “C” for the mother’s level of comfort, and “H” for the amount of support the mother has be given to hold her infant to the breast. Each parameter is scored using a numerical score of 0, 1, or 2 [19]. The LATCH scale was designed to assess the success of breastfeeding in this study since it is a useful tool in mother-infant pairs who might benefit from additional skilled support to initiate breastfeeding in specific subgroups at risk of non-exclusive breastfeeding at discharge [21].

The “L” assessment was scored as “2” if good latching was identified (grasps breast, tongue down, lips flanged and rhythmic sucking); “1” if repeated attempts to hold the nipple in the mouth or to stimulate to suck were identified, and “0” if poor latching (too sleepy or reluctant or no latching achieved) was seen. The “A” assessment was scored as “2” if audible swallowing occurred (spontaneous and intermittent < 24 h old or spontaneous and frequent > 24 h old), “1” if a few swallows occurred with stimulation, and “0” if ineffective swallowing occurred. The “T” assessment was scored as “2” if an everted nipple was present (after stimulation), “1” if the nipple was flat, and ‘0’ if the nipple was inverted. The ‘C’ assessment was scored as “2” if the breast was soft and tender, “1” if the breast was filled or reddened / featured small blisters / bruised nipples, and “0” if the breast was engorged or if a crack appeared. The ‘H’ assessment was scored as “2” if good positioning was achieved (no assistance from the staff or mother able to position / hold infant), “1” if minimal assistance was required (i.e., elevate the head of the bed or place pillows for support), and “0” if full assistance was required (staff held the infant at the mother’s breast) [19]. The total score ranges from 0 to 10, with the higher score representing efficient breastfeeding techniques. A total score of more than 7 is regarded as successful breastfeeding, and a score of less than 7 is considered as unsuccessful breastfeeding [19].

### Method of data collection

The midwives who worked regularly in the birthing suite agreed that the researcher could attend and record observations of consenting mothers while they were being provided with care. The midwives were requested to behave as if the researcher was not present and not to make changes to their normal practice. The researcher arrived at the delivery room, confirmed the consent of the laboring woman and her relatives, and gained her presence permission from the person managing the birth. The observation equipment included the observation record sheet on a clipboard, a stopwatch, a thermometer and a pen. When birth was imminent, the researcher entered the room to observe. At the moment of birth, the researcher started the stopwatch to record the time following the birth. The researcher stayed with each woman until the end of the first hour after birth.

In the routine care group, the infant was delivered by a midwife, wrapped in blankets, placed under a warmer, and then dried. The Apgar score was determined immediately after the umbilical cord was cut. The infants were provided with this routine care by the midwife working in the delivery room. After the infants were weighed, dressed, and measured, they were handed to their mothers who were encouraged to begin breastfeeding. The routine care of placing a newborn under a warmer is performed in the least time possible (4–5 min) in Hawler maternity teaching hospital due to the presence of only two warmers in the birthing suite for a five-bed room, which are almost always occupied.

With the assistance of the researcher, infants in the intervention group were placed undressed in a prone position against their mothers’ bare chest between breasts immediately after birth and before placental delivery or suturing of tears or episiotomy. The Apgar score was determined, the infant’s nose and mouth were suctioned while on the mother’s chest, the infant was dried, and both mother and infant were covered with a pre-warmed blanket. To prevent heat loss, the infant’s head was covered with a dry cap that was replaced when it became damp. Dressing and measuring of the infant were postponed to one hour after the delivery by a registered midwife.

By standing behind or next to the bed and approaching closer to view the actions, the researcher monitored the infants while they were exhibiting feeding behaviors such as mouthing, licking, latching, and suckling. Breastfeeding initiation time after birth and duration of the first breastfeed were recorded, and then the LATCH scale was used to assess the success of the first breastfeed in the two groups. Some of the mothers in the two groups asked the researcher for assistance to breastfeed their newborns; therefore, the degree of assistance provided by researcher was scored along with other parameters of the LATCH scale (latch, audible swallowing, nipple type, comfort).

Active management of the third stage of labor was performed for all participants by a registered midwife. This composed of three steps: 1) administration of 10 IU synthetic oxytocin, immediately after birth of the baby; 2) controlled cord traction (CCT) to deliver the placenta; and 3) massage of the uterine fundus after the placenta is delivered [18]. The researcher did not interfere with the delivery of the placenta and just observed this procedure being performed by the midwife. Duration of the third stage of labor, which starts with the delivery of the fetus and end with the complete delivery of the placenta was measured by the researcher [18].

In the 1991 World Health Organization (WHO) guidelines it was recommended that rectal temperature should be limited and axillary temperature should be used routinely for the newborn [22]. Therefore, in this study, axillary temperature of the newborns in both groups was checked 30 min after birth. The measuring range of the thermometer was 32–42 °C with accuracy to the nearest tenth of a degree. The thermometer sensor was sterilized with 70% alcohol before each use. After the button power was activated, the digital thermometer was turned on and put with the sensor in the newborn’s armpit, and kept there until the alarm sound was heard. The score on the screen showed the measured body temperature. Based on WHO’s guidelines (1991), an axillary temperature of less than 36.0 °C in newborns is considered as hypothermia [23].

### Statistical methods

Data were analyzed using SPSS statistical analysis software. Descriptive relationships between demographic variables and type of care provided for mothers and newborns after birth were explored using means and standard deviations (SD) for continuous variables, whilst categorical variables were described using proportions. The relationship between SSC and time to initiate breastfeeding, duration of third stage of labor, success of breastfeeding, newborn hypothermia, and temperature of the newborn 30 min after birth were analysed using T tests and Chi square tests. Logistic regression modelling was used to examine the effect of SSC and conventional care on outcomes of the study by adjusting for potential confounders like mother’s age, education level, occupation, parity, and newborn gender. The level of statistical significance was set at *p* <  0.05 in this study. This study had 100% power at a 95% level of confidence to detect 38 and 56% difference in initiation of the breastfeeding and newborn temperature between mother-newborn who experienced SSC and mother-newborns who underwent routine care. The equivalent power value to detect 17% differences in duration of third stage of labor was 81%.

## Results

This study was carried out on 108 mothers and their newborns. The results showed that mean age of the mothers in the SSC and routine care groups was 26.29 **±** 6.13 (M **±** SD) and 26.02 **±** 5.94 (M **±** SD) respectively. Higher numbers of the mothers in the routine care group had secondary and academic education compared with mothers in the intervention group. Most of the mothers in both groups were non-employed and multigravid. Approximately, the same proportion (50%) of the mothers in both groups were primipara. The results showed that the two groups were not significantly different in terms of the mothers’ demographic characteristics including maternal age, occupation, gravidity, number of miscarriages, parity and number of antenatal visits (Table 1). This study revealed that 48% versus 46% of the newborns who experienced SSC or routine care achieved successful breastfeeding, respectively (Table 2).

**Table 1:** Maternal and newborn characteristics in SSC and routine care groups

	SSC group (*n* = 56)	Routine care group (*n* = 52)	*P* value*
	n (%)	n (%)	
- Maternal age			
• < 18 • 18–35 • > 35 • Mean ± SD	2 (4) 48 (86) 6 (11) 26.29 ± 6.13	5 (10) 43 (83) 4 (8) 26.02 ± 5.94	0.4
- Mother’s education level			
• Illiterate • Primary school • Secondary school • Academic education	24 (43) 24 (43) 3 (5) 5 (9)	18 (35) 15 (29) 12 (23) 7 (13)	0.03
- Mother’s occupation			
• Non employed • Employee	50 (89.2) 6 (10.71)	46 (88) 6 (11)	0.6
- Gravidity			
• Primigravid • Multi gravid (2–4)	13 (23.2) 43 (76.8)	12 (23) 40 (77)	0.9
- Number of miscarriage			
• 0 • 1–2	45 (80.4) 11 (19.7)	42 (81) 10 (17)	0.7
- Parity			
• 0–1 • 2–3 • 4	30 (54) 20 (36) 6 (11)	26 (50) 16 (31) 10 (19)	0.4
Number of antenatal visit			
• < 3 • > 3	49 (87) 7 (12)	46 (88) 6 (11)	0.8
- Gender of newborns			
• Male • Female	25 (44) 21 (55)	25 (48) 27 (52)	0.7

- Data are presented as mean (standard deviation) in case of continuous variables and n (%) in case of frequencies

*Chi-square test

**Table 2:** Success of breastfeeding in mothers with skin-to-skin contact and routine care

Item	Score	Skin-to-skin contact group (*n* = 56)	Routine care group(*n* = 52)	*p*-value *
		n (%)	n (%)	
Latch	• (0) Too sleepy or reluctant, not latch obtained	1 (2)	0 (0%)	0.62
	• (1) Repeated attempts, must hold nipple in mouth, must stimulate to suck	30 (54%)	29 (56%)	
	• (2) Grasps breast, tongue down and forward, lips flanged, Rhythmic suckling	24 (43%)	23 (44%)	
Audible swallowing	• (0) None • (1) A few with stimulation • (2) Spontaneous and intermittent	0 (0%) 26 (46%) 30 (54%)	3 (6%) 21 (40%) 28 (54%)	0.17
Type of nipple	• (0) Inverted • (1) Flat • (2) Everett after stimulation	0 (0%) 9 (16%) 47(84%)	0 (0%) 18 (35%) 34 (65%)	0.02
Comfort	• (0) Engorged, cracked, bleeding, large blister, severe discomfort.	0 (0%)	0 (0%)	
	• (1) Filling, Reddened, small blister or bruises, mother complains, mild/moderate discomfort	6(11%)	6 (11%)	
	• (2) Soft, non- tender, intact nipple	50 (89%)	46 (88%)	0.89
Hold	• (0) Full assist (staffs holds the baby at breast)	13 (23%)	19 (36%)	0.24
	• (1) Minimal assistant, Teach one side mother - does other staff holds and then mother take over	23 (42%)	20 (38%)	
	• (2) No assist from staff, mother able to position and hold the infant	20 (36%)	12 (23%)	
Total score	• > 7 (Successful breastfeeding) • < 7 (Unsuccessful breastfeeding)	27 (48%) 29 (52%)	24 (46%) 28 (54%)	0.83

Note: LATCH scale [20] was used in this study to assess success of breastfeeding

*Chi square test

There was an association between mother and newborn SSC and breastfeeding initiation time after birth. Newborns who experienced SSC initiated breastfeeding 2.41 ± 1.38 (M ± SD) minutes after delivery, while newborns in the routine care group started breastfeeding 5.48 ± 5.70 (M ± SD) minutes following their birth (*p* <  0. 001).

As shown in Table 3, duration of the first breastfeed in mothers with SSC versus routine care was 23.07 ± 7.89 (M ± SD) and 23.79 ± 8.22 (M ± SD) respectively; however, this difference was not statistically significant. In mothers who practiced SCC, the duration of the third stage of labor was significantly shorter than that of the control group (6 ± 1.74 (M ± SD) versus 8.02 ± 3.69 (M ± SD) minutes) (*p* <  0.001). The average axillary temperature of the newborns who experienced SSC was 37.33 ± 0.65 °C, while it was 36.18 ± 0.99 °C in newborns in the routine care group. It was found that 98% of the newborns in the SSC group had normal temperature and 2% of them had hypothermia. On the other hand, 42% the newborns in the routine care group had hypothermia.

**Table 3:** Comparison of breastfeeding behaviors, newborn temperature and third stage of labor between groups

Items	Skin-to-skin contact group (*n* = 56)	Routine care group (*n* = 52)	*p* value
- Time to initiate breastfeeding (minute)			
• (Mean ± SD) • Range	2.41 ± 1.385 1–9	5.48 ± 5.704 1–19	< 0.001^a^
- Duration of first breastfeed(minute)			
• (Mean ± SD) • Range	23.07 ± 7.89 10–35	23.79 ± 8.22 10–25	0.64^a^
- Temperature of newborns (degree Celsius)			
• (Mean ± SD) • Range	37.33 ± 0.65 35.62–38.50	36.18 ± 0.99 33.5–38.1	< 0.001^a^
- Newborn hypothermia			
• Yes • No	1 (2%) 55 (98%)	30 (58%) 22 (42%)	< 0.000^b^
- Duration of third stage of labor (minute)			
• (Mean ± SD) • Range	6 ± 1.7 1–10	8.02 ± 3.6 1–20	< 0.001^a^

^a^Chi square test

^b^T test

After using the regression model for adjustment of potential factors and background characteristics such as age, education level, employment, parity and newborn gender, there was an association between newborn temperature [Odds Ratio (OR) 0.01; 95% Confidence Interval (CI) 0.002, 0.12] newborn hypothermia (OR 180.3; 95% CI 3.84, 8480), time to initiate breastfeeding (OR 2.86; 95% CI 1.68, 4.85) and duration of third stage of labor (OR 2.14; 95% CI 1.27, 3.6) with SSC (Table 4).

**Table 4:** Adjusted relationship of breastfeeding behaviors, newborn temperature and third stage of labor with SSC

Items	Odd Ratio (95% Confidence Interval)	*p*-value*
- Maternal age	0.93 (0.76, 0.1)	0.53
- Maternal education	2.5 (0.98, 11)	0.06
- Occupation	0.69 (0.01, 27.04)	0.84
- Number of para	2 (0.92, 4.71)	0.07
- Newborn gender	0.22 (0.04, 1.19)	0.08
- Time to initiate breastfeeding	2.86 (1.68, 4.85)	< 0.001
- Duration of first breastfeed	0.96 (0.86, 1.08)	0.53
- Duration of third stage of labor	2.14 (1.27, 3.6)	0.004
- Successful breastfeeding	0.8 (0.55, 1.17)	0.26
- Newborn temperature	0.01 (0.002, 0.12)	< 0.001
- Newborn hypothermia	180.3 (3.84, 8480)	0.008

*Logistic regression

## Discussion

Fifty-two percent of the women in this study received mother-newborn SSC immediately after birth, and 48% experienced convention care after delivering their baby. In this study, mothers in the SSC group had completed lower levels of education compared to mothers in the routine care group; however, this difference was not associated with the outcomes assessed in this study when logistic regression analysis was applied. No relationship was observed between other maternal characteristics with SSC and routine care.

According to the results of the present study, contact through the skin between the women and their newborns after birth led to greater initiation of breastfeeding. It is not clear why SSC improved breastfeeding behaviors of healthy full term infants, however, similar findings have been reported in the literature [24]. The American College of Nurse-Midwives state that SSC helps infants smell and find the nipple so that breastfeeding will be initiated by them more rapidly and successfully [25]. This can be attributed to the high levels of catecholamine immediately after birth, which makes olfactory bulbs in the infant’s nose extremely sensitive to odor cues [26]. The results of the studies carried out by Moore and Anderson in USA [27], Khadivzadeh and Karimi in Iran [28], and Mahmood et al. in Pakistan [29] showed that early contact improved breastfeeding initiation and prolonged the duration of breastfeeding in infants. Early initiation of breastfeeding stimulates breast milk production, produces antibody protection for the newborn and its practice determines the successful establishment, longer duration of breastfeeding, and lower risk of neonatal mortality [30].

In their study, Essa et al., using the Infant Breastfeeding Assessment Tool (IBFAT), found that the SSC and control groups were statistically different in terms of the success of the first breastfeed rate [31]. However, the LATCH scale used in this study to assess the success of breastfeeding found no statistical difference between the two groups. This discrepancy could be due to a difference in the tools used to assess the success of breastfeeding.

Neonatal hypothermia is an important contributing factor to neonatal mortality and morbidity in both developed and developing countries; especially in developing countries [10]. The Maternal and Child Health Program by the WHO has issued guidelines for prevention of neonatal hypothermia as one of the elements of essential care in the newborn at birth and on the first day of life [32]. In the present study, 42% of the newborns who did not receive SSC care had hypothermia; however, just 2% of the newborns who received SSC developed hypothermia after birth. In their study on 160 term neonates in Iran, Keshavarz and Haghighi investigated the effects of kangaroo contact on physiological variables after cesarean section. In so doing, the neonates were randomly assigned into a SSC group and a routine care group. The newborns’ temperatures in both groups were measured half an hour after the cessation of contact. The mean temperature was significantly different in the SSC and routine care groups (36.8 °C and 36.6 °C respectively), and the mean temperature one hour after SSC was 36.9 °C, which was 0.3 °C higher than the mean temperature in the control group (푃 = 0.001) [33]. The results of a meta-analysis comprised of 23 studies indicated strong evidence of increased body temperature as a result of SSC. It is interesting to note that the ambient temperature did not influence the outcome of body temperature, as even in colder environments the body temperature of newborns who received SCC increased or at least remained unchanged [34]. Transfer of heat from the mother to the newborn facilitated by direct skin contact has been demonstrated to be at least as effective as incubator care for warming [35].

Assessing the effect of SSC on duration of the third stage of labor in the present study showed that the mothers who had SSC with their infant after birth had a shorter third stage in comparison with those who received routine care (6 min vs 8.02 min). Similar findings have been reported in a study carried out in Baghdad, Iraq by Mejbel and Ali to examine the effectiveness of SSC on the duration of the third stage of labor [36]. In an Egyptian investigation of low risk primiparous women who either received SSC or routine hospital care, the mean duration of the third stage of labor in the SSC group was significantly shorter (2.8 ± 0.85 min) than the routine care group (11.22 ± 3.33 min) (*p* < .01) [31]. Common practices used in the management of third stage of labor neither facilitate the production of a mother’s own oxytocin nor reduce catecholamine levels during the first minutes after birth, both of which can be expected to physiologically improve the new mother’s contractions and thus reduce her blood loss. The routine practice of separating mother and infant deprives the mother of important opportunities to increase her natural oxytocin levels [12].

The results of the present study need to be considered in the light of its limitations. In this study, there were no data on exclusive breastfeeding, and duration of breastfeeding was not assessed. In future studies, it would be beneficial to look at exclusive breastfeeding after discharge through a longitudinal study. The researcher of the present study was faced with some challenges in facilitating SSC for the mothers in the intervention group. This may have been because mothers in this study had low knowledge regarding SSC care, as a result of poor provision of health education at antenatal care units in primary health care center in Erbil: only 23.7% of visiting women receive education about infant care and breastfeeding [15]. Increased workload in the obstetric unit did not allow the researcher to continue SSC more than one hour after birth, although most of the mothers were very pleased and enjoyed the experience of SSC and wished to prolong its duration.

## Conclusion and recommendations

To reduce the current prevalence of high neonatal morbidity and mortality rate in Iraq, there is a dire need for simple and cost-effective prevention and (complementary) intervention methods that are easily accessible to mothers and can be applied immediately after birth. Mother and newborn SSC is a low-cost intervention that would be accessible, simple, and feasible for most mothers in developing countries. In order to accomplish this goal, the old paradigms of labor and delivery care need to be changed and immediate, uninterrupted SSC after birth should be practiced. Unlimited opportunities for SSC and breastfeeding promote optimal maternal and child outcomes. It is critical to provide all midwives in delivery rooms with continuous educational and training programs on how to implement SSC for all mothers. These changes directly support the millennium goals of improved maternal and child health.
